# Exosome Therapy in Stress Urinary Incontinence: A Comprehensive Literature Review

**DOI:** 10.3390/biomedicines13051229

**Published:** 2025-05-19

**Authors:** Manouchehr Nasrollahzadeh Saravi, Mahdi Mohseni, Iman Menbari Oskouie, Jafar Razavi, Ernesto Delgado Cidranes, Masoumeh Majidi Zolbin

**Affiliations:** 1 Children’s Medical Center, Tehran University of Medical Sciences, Tehran 1419733151, Iran; manouchehrnasr@gmail.com (M.N.S.); mmmhdimh@gmail.com (M.M.); 2 Urology Research Center, Tehran University of Medical Sciences, Tehran 1419733151, Iran; imanmenbary@gmail.com; 3 Vali-E-Asr Reproductive Health Research Center, Family Health Research Institute, Tehran University of Medical Sciences, Tehran 1419733141, Iran; dr.razavishirazi@gmail.com; 4 Pain Management Department, University Hospital Vithas Madrid La Milagrosa, 28010 Madrid, Spain; edelgadocidranes@gmail.com; 5 Pediatric Urology and Regenerative Medicine Research Center, Gene, Cell & Tissue Research Institute, Children’s Medical Center, Tehran University of Medical Sciences, Tehran 1419733151, Iran

**Keywords:** exosome therapy, stress urinary incontinence, regenerative medicine, extracellular vesicles

## Abstract

Stress urinary incontinence (SUI) is characterized by the involuntary leakage of urine when bladder pressure exceeds urethral closing pressure during routine activities such as physical exertion, coughing, exercise, or sneezing. SUI is the most prevalent form of urinary incontinence, with a reported prevalence ranging from 10% to 70%, and its incidence increases with age. As the global population continues to age, the prevalence and clinical significance of SUI are expected to rise accordingly. The pathophysiology of SUI is primarily driven by two mechanisms: urethral hypermobility, resulting from compromised supporting structures, and intrinsic urethral sphincter deficiency, characterized by the deterioration of urethral mucosa and muscle tone. Current treatment options for SUI include conservative management strategies, which heavily rely on patient adherence and are associated with high recurrence rates, and surgical interventions, such as sling procedures, which offer effective solutions but are costly and carry the risk of adverse side effects. These limitations highlight the urgent need for more effective and comprehensive treatment modalities. Exosomes, nano-sized (30–150 nm) extracellular vesicles secreted by nearly all cell types, have emerged as a novel therapeutic option due to their regenerative, anti-fibrotic, pro-angiogenic, anti-apoptotic, anti-inflammatory, and anti-hypoxic properties. These biological functions position exosomes as a promising alternative to conventional therapies for SUI. Exosome therapy has the potential to enhance tissue regeneration, restore urethral function, and repair nerve and muscle damage, thereby reducing symptom burden and improving patients’ quality of life. Additionally, exosome-based treatments could offer a less invasive alternative to surgery, potentially decreasing the need for repeated interventions and minimizing complications associated with current procedures. In this literature review, we critically assess the current state of research on the potential use of exosomes in treating SUI, highlighting their therapeutic mechanisms and potential clinical benefits.

## 1. Introduction

Urinary continence results from the synergistic action of multiple mechanisms that prevent unintentional urinary leakage. These mechanisms include neurogenic factors, urethral structures, muscles, and connective tissue. Impairments or dysfunction in these components can result in involuntary urine leakage, commonly referred to as incontinence [1]. There are several types of incontinence, with stress incontinence being the most common. Stress incontinence is defined as the involuntary leakage of urine when bladder pressure exceeds urethral closing pressure during routine daily activities such as physical exertion, coughing, exercise, and sneezing [2,3]. This condition has adverse consequences, affecting physical, social, and psychological well-being. Previous studies have linked stress urinary incontinence (SUI) to falls and fractures, sleep disturbances, and urinary tract infections, resulting in impaired quality of life and self-isolation, hindering patients from achieving even simple daily goals [4].

Risk factors for SUI include female sex, pregnancy and delivery, history of pelvic surgery, smoking, chronic cough, obesity, and a history of bladder or prostate cancer. This condition affects a significant number of individuals worldwide, with prevalence rates ranging from 10% to 70% depending on the population and study methods. In the United States, from 2017 to 2020, approximately 46% of adult women were affected by SUI [5]. Although it mostly occurs in women, a 31% prevalence rate has been reported in men with a history of prostate or bladder cancer [6]. The incidence of SUI increases with age, and as the global population continues to age, the prevalence and associated management costs of SUI are projected to rise significantly in the coming years. Current treatment options for SUI have significant limitations. Conservative approaches, such as lifestyle modifications, pelvic floor physiotherapy, and the use of bulking agents, rely heavily on patient adherence and resilience. These methods may be effective in short-term; however, with a reported long-term success rate of only 43% [7], they often require repeated treatments and ongoing lifestyle adjustments, such as managing fluid intake and avoiding activities that exacerbate leakage. Surgical interventions, including mid-urethral slings, offer higher cure rates (up to 80%) but are associated with substantial costs and risks, such as infections, urinary retention, fistula formation, chronic pain, sexual dysfunction, and tape erosion or extrusion. These complications may lead to additional surgeries, underscoring the critical need for innovative, effective, and minimally invasive treatment alternatives [4,8,9].

In the past decade, regenerative medicine has rapidly advanced, with stem cells at its forefront. These cells have shown significant potential in treating the underlying causes of various diseases. However, as living cells, they present challenges such as immunogenicity, tumorigenicity, high costs, ethical concerns, low survival rate of cells, and limited supply [10]. Consequently, researchers have turned their attention to the paracrine effects of stem cells, leading to the discovery of exosomes [11]. Remarkably, exosomes exhibit similar behaviors and characteristics to their parent stem cells.

Exosomes are small, nano-sized extracellular vesicles with diameters ranging from 30 to 150 nm. They contain various components, including lipids, nucleic acids, and RNAs, and play a crucial role in cell-to-cell communication through surface receptor molecules and ligands. These vesicles are present in nearly all cells, tissues, and body fluids [12]. Unlike stem cells, exosomes do not carry risks associated with living cells, such as immunological rejection. Additionally, they can be freeze-dried and stored as shelf-stable products, enhancing their accessibility and cost-effectiveness [13]. Moreover, due to their nanoscale dimensions, exosomes can be sterilized through filtration. Additionally, their administration is versatile, extending beyond intramuscular and intravenous routes to include localized delivery methods such as aerosol sprays, subcutaneous injections, and intrathecal therapy. Furthermore, exosomes can be bioengineered to display specific surface ligands for targeted therapy and carry selected proteins, nucleic acids, and drugs, thereby enhancing their therapeutic potential [14,15].

Exosomes have demonstrated regenerative, anti-fibrotic, pro-angiogenic, anti-apoptotic, anti-inflammatory, and anti-hypoxic properties, making them promising in addressing various pathological conditions. They have shown exciting potential in treating cancer, cardiovascular diseases, reproductive disorders, liver fibrosis, neurodegenerative diseases, and musculoskeletal conditions. This capability allows scientists and clinicians to target diseases at a cellular level, offering new avenues for therapeutic interventions [11].

Research on exosome therapy for SUI has gained traction in recent years, as existing treatment methods often fail to significantly enhance patients’ quality of life [16]. Exosomes, due to their critical role in cell communication and tissue repair, present a promising therapeutic option for SUI. Furthermore, the identification of biomarkers and patient-specific factors that influence treatment outcomes can pave the way for the development of personalized, less invasive therapies, ultimately improving patient outcomes and quality of life.

In this literature review, we aim to consolidate current knowledge on the application of exosome therapy on SUI, providing a comprehensive overview of the current state of research. To achieve this, we conducted a thorough search of PubMed, Google Scholar, and ClinicalTrials.gov to identify relevant studies. This review highlights promising findings, identifies gaps in the existing knowledge, and suggests areas for future investigation.

## 2. Pathophysiology of SUI

SUI is defined by the involuntary leakage of urine during activities that increase intra-abdominal pressure, such as coughing, sneezing, or exercise. This condition arises when the mechanisms responsible for maintaining urinary continence fail. The two primary mechanisms implicated are urethral hypermobility and intrinsic urethral sphincter deficiency. Urethral hypermobility occurs when the urethra experiences excessive movement due to weakened support structures, leading to improper closure during increased abdominal pressure. urethral sphincter deficiency refers to the inability of the urethral sphincter to function effectively, resulting in urine leakage even without increased abdominal pressure. The pathophysiology of SUI is multifactorial, involving pelvic floor dysfunction, neural damage, and hormonal influences. Collectively, these factors impair the lower urinary tract’s ability to withstand bladder pressure, resulting in urine leakage [4]. Below is a summarized exploration of these mechanisms.

### 2.1. Inflammation and Oxidative Stress

Chronic inflammation plays a pivotal role in the deterioration of pelvic floor integrity. Factors like mechanical stress, pregnancy, aging, and hormonal fluctuations can cause tissue micro trauma, initiating an inflammatory response. This response involves the release of pro-inflammatory cytokines, including tumor necrosis factor-alpha (TNF-α) and interleukin-6 (IL-6), leading to extracellular matrix (ECM) remodeling, fibrosis, and apoptosis of muscle and nerve cells, thereby impairing urethral function [17].

Oxidative stress arises from an imbalance between reactive oxygen species (ROS) production and the body’s antioxidant defenses. In SUI, elevated ROS levels contribute to mitochondrial dysfunction, DNA damage, and lipid peroxidation, exacerbating muscle atrophy and fibrosis. Aging, ischemia, and mechanical strain on pelvic tissues further increase oxidative stress, compromising muscle contractility and urethral sphincter function. In sum, inflammation and oxidative stress are central to the development of SUI as they promote tissue degradation and dysfunction [18].

### 2.2. Pelvic Floor Dysfunction

The pelvic floor supports the bladder and urethra, and its integrity is crucial for maintaining continence. Pelvic floor dysfunction in SUI can result from the following:

#### 2.2.1. Muscle Damage

##### Myostatin and TNF-α

These are key regulators of muscle growth that impact SUI. Myostatin inhibits muscle cell proliferation and differentiation by activating the Smad2/3 pathway, leading to reduced muscle thickness and function. Various factors, such as aging, hormonal imbalances, persistent inflammation, physical inactivity, neuromuscular impairments, and metabolic conditions, can contribute to elevated myostatin levels in SUI.

Reducing myostatin levels using CRISPR interference has shown promise in improving urethral muscle function in SUI models. TNF-α induces muscle cell apoptosis, particularly in the elderly, and suppresses the differentiation of urethral rhabdosphincter progenitor cells via the PI3K and p38-MAPK pathways. TNF-α antagonists may counteract these effects, offering a potential therapeutic approach for managing SUI [19,20,21].

##### Anoctamin-1 (ANO1)

A calcium-activated chloride channel is crucial for urethral smooth muscle contraction and maintaining spontaneous muscle tone. This function, especially important in females, helps with urinary retention and reduces urine leakage.

A decline in ANO1 levels in SUI may be caused by factors such as aging, hormonal imbalances, persistent inflammation, oxidative stress, neuromuscular impairments, physical inactivity, and metabolic conditions. In mice lacking ANO1 in smooth muscle cells, urethral closure mechanisms are notably enhanced [21].

##### Vitamin D

Vitamin D is vital for calcium regulation, bone health, and muscle function. Deficiency in this vitamin, especially in older adults, can lead to reduced muscle mass and strength, particularly affecting fast-twitch muscle fibers, which are critical for quick contractions. This weakness can impair urethral closure during strain, increasing the risk of SUI. Additionally, adequate prenatal vitamin D levels are linked to stronger postpartum pelvic floor muscles, further emphasizing its importance in preventing SUI [20].

#### 2.2.2. Connective Tissue Damage

The effective closure of the female urethra relies on the coordinated action of various anatomical structures, including connective tissue, pelvic fascia, ligaments, and the anterior vaginal wall. These structures play a pivotal role in maintaining the mechanical stability of the genitourinary tract by supporting the bladder neck, urethra, and pelvic organs. Defects or weaknesses in connective tissue can lead to the relaxation of these structures, impeding urethral closure and resulting in SUI symptoms. Reduced collagen and elastin levels in ECM lead to weakened urethral support. Imbalances in matrix metalloproteinases (MMPs) and their inhibitors, TIMPs (tissue inhibitors of metalloproteinase), further degrade the ECM, exacerbating tissue weakness [4].

### 2.3. Neural Damage

Urinary continence relies on intact neural control of the bladder and urethral sphincters. Neural damage, particularly to the pudendal nerve, disrupts these pathways. This damage may result from childbirth, surgery, or trauma, impairing the external urethral sphincter’s function and leading to involuntary urine leakage [22].

### 2.4. Hormonal Influences

Hormones such as estrogen and testosterone regulate pelvic floor tissues. Declining estrogen levels in postmenopausal women reduce collagen synthesis and muscle tone, while lower androgen levels contribute to reduced pelvic muscle strength [23].

## 3. Current Treatments and Their Challenges in SUI

Understanding the underlying mechanisms and contributing factors of SUI is essential for selecting appropriate treatment options. Current treatment options for SUI are diverse and encompass conservative measures, device-based therapies, and surgical interventions, each presenting specific benefits and limitations which is gathered in Table 1:

Despite numerous treatment options for SUI, each has inherent limitations, often due to a lack of focus on the underlying pathophysiology, such as damage to the pubococcygeus muscle and pudendal nerve. The pubococcygeus muscle, part of the levator ani group, supports the bladder, uterus, and rectum, and it plays a key role in maintaining urinary continence by controlling urethral closure during increased intra-abdominal pressure (e.g., coughing or sneezing). Damage or weakening of this muscle, caused by trauma, childbirth, aging, or certain medical conditions, reduces urethral support, increasing SUI risk. The pudendal nerve, originating from the sacral plexus (S2–S4), controls the external urethral sphincter and pelvic floor muscles, including the pubococcygeus. Damage to this nerve from childbirth, pelvic surgeries, or chronic straining can impair pelvic floor muscle function, reducing muscle tone and strength and contributing to SUI. Unlike other current treatment options, exosome therapy holds promise in the regeneration and repair of the pudendal nerve, connective supportive tissue, and pubococcygeus muscle, targeting the specific underlying pathologies leading to SUI, thus offering a novel and potentially transformative approach to SUI management [4,36].

## 4. Exosome Biology and Therapeutic Potential

### 4.1. Overview on Exosomes

Multicellular organisms rely on intercellular communication, which occurs through direct cell-to-cell contact or the transfer of secreted molecules like cytokines or chemokines. Previously, researchers believed these were the primary modes of communication. However, in the past three decades, a new form of intercellular communication has been identified: extracellular vehicles (EVs). Exosomes, nano-sized EVs (30–150 nm), are secreted by nearly all cell types and have been detected in various biological fluids, including blood, urine, and breast milk. Originally thought to be cellular waste disposal systems, exosomes are now recognized as pivotal mediators of intercellular communication and regulators of tissue homeostasis. These vesicles are enriched with bioactive molecules such as proteins, lipids, and nucleic acids, which they transfer to recipient cells, influencing cellular behavior and function [37,38,39,40,41,42,43,44,45,46].

These features drew the attention of scientists to the therapeutic potential of exosomes.

### 4.2. Exosome Biogenesis and Composition

Suntres et al. described exosomes as cytoplasm encapsulated by a lipid bilayer with external domains of transmembrane proteins [47]. Exosome biogenesis occurs within multivesicular bodies (MVBs), where intraluminal vesicles are formed through the inward budding of endosomal membranes. MVBs either fuse with the plasma membrane to release exosomes or are directed to lysosomes for degradation [48,49,50]. The two major pathways involved are the ESCRT-dependent and independent pathways [49,51,52,53,54]. Understanding these pathways is key to better comprehending the composition, function, and effects of exosomes.

Exosomes are nanoscale extracellular vesicles that encapsulate a diverse array of biomolecules, including proteins, lipids, and nucleic acids such as mRNA and microRNA (miRNA). Comprehensive analyses have identified approximately 4400 proteins, 194 lipids, 1639 mRNAs, and 764 miRNAs within exosomes, underscoring their complexity and multifaceted roles in cellular functions [55]. Exosomes are characterized by a lipid bilayer membrane enriched in cholesterol, sphingomyelin, and ceramide, which not only stabilize the membrane but also participate in signaling processes. Their protein composition includes both proteins, such as tetraspanins (e.g., CD9, CD63, CD81) and heat shock proteins (e.g., HSP70, HSP90), which are found in almost all exosomes regardless of their parent cell, as well as proteins specific to their cells of origin. Notably, exosomes secreted by prostate cancer cells contain prostate-specific antigens, presenting potential diagnostic applications. Similarly, urinary exosomes have demonstrated significant diagnostic potential. Additionally, exosomes carry various forms of RNA, including mRNA, miRNA, and long non-coding RNA. The specific composition of exosomes is influenced by the cell type of origin and its physiological or pathological state. Consequently, exosomes secreted by mesenchymal stem cells carry the regenerative and proliferative potential of these cells without the risks associated with cellular therapy. This diverse cargo enables exosomes to participate in various cellular processes, including gene regulation, protein synthesis, and membrane repair [13,56,57].

### 4.3. Exosome Action and Role in Tissue Repair and Regeneration

Tissue regeneration depends heavily on a cascade of events and interaction between various cells. Exosomes have gained considerable attention in regenerative medicine due to their ability to mimic the therapeutic effects of parent cells without the associated risks, such as tumorigenicity and immune rejection. Unlike stem cells, exosomes can be stored, handled, and delivered more efficiently, making them a versatile tool for therapy.

The regenerative properties of exosomes have been demonstrated in numerous tissues, including muscle, nerve, and ECM. Their ability to modulate inflammation, enhance angiogenesis, and promote cellular proliferation and differentiation makes them particularly relevant for conditions like SUI, which involve damage to multiple tissue types. [58,59].

## 5. Relevance of Exosomes in SUI Therapy

SUI results from weakened pelvic floor muscles, nerve damage, and ECM degradation. Exosomes offer a multifaceted therapeutic approach by targeting the following underlying mechanisms:

### 5.1. Nerve Repair

Nerve damage, particularly involving the pudendal nerve, is a significant contributor to SUI. Exosomes derived from Schwann cells (SCs) and mesenchymal stem cells have been shown to promote nerve regeneration by activating pathways such as Wnt/β-catenin and PI3K-Akt. These pathways enhance SC proliferation, inhibit apoptosis, and support axonal repair. For instance, SC-derived exosomes have demonstrated protective effects on damaged dorsal root ganglion cells in preclinical models, highlighting their potential to restore nerve function in SUI [60,61,62,63]. Following nerve injuries, several mechanisms are activated to promote regeneration, including Wallerian degeneration, recruitment of macrophages to remove debris, and the critical involvement of SCs [64,65]. Notably, the impact of exosomes on these mechanisms has been reported in studies. These vesicles can activate and regulate the differentiation and proliferation of SCs while preventing apoptosis in these cells [66,67,68,69]. Consequently, SCs enhance nerve differentiation and proliferation and produce neurotrophic factors that promote nerve growth, leading to the regeneration of injured nerve tissue. Moreover, exosomes can directly upregulate neurotrophic factors such as glial cell-derived neurotrophic factor, fibroblast growth factor-1, brain-derived neurotrophic factor, insulin-like growth factor-1, and nerve growth factor, further promoting tissue repair [70]. Lastly, their immunomodulatory effects are significant in nerve repair, as they upregulate the M2 macrophage population, known for its anti-inflammatory effects, thus creating a supportive environment for nerve regeneration [58].

### 5.2. Muscle Regeneration

The pubococcygeus muscle, vital for urinary continence, often exhibits atrophy in SUI patients. Exosomes can activate satellite cells, a type of muscle stem cell, to promote muscle repair. Studies have shown that exosomes from urine-derived stem cells enhance the expression of myogenic markers, such as Pax7 and MyoD, facilitating the formation of new muscle fibers [71]. Moreover, exosomes can modulate the inflammatory environment by polarizing macrophages toward an M2 phenotype, creating conditions favorable for muscle regeneration [72,73,74,75,76]. Several mechanisms are involved in myogenesis, including angiogenesis, activation and differentiation of satellite cells, and immunomodulation. Achieving effective myogenesis requires the activation of muscle-specific transcription factors and genes, with miRNAs playing a fundamental role in this process. Exosomes, which are rich in miRNAs, can induce myogenesis by transferring these essential components [77,78]. These particles can enhance angiogenesis, improve capillary density [79,80], and induce the differentiation of satellite cells into myotubes and myofibers [73,81], ultimately leading to muscular regeneration.

### 5.3. Anti-Inflammatory and Oxidate Stress Effects

Chronic inflammation exacerbates tissue damage in SUI. Exosomes exhibit potent immunomodulatory properties by decreasing pro-inflammatory cytokines (e.g., TNF-α, IL-1β) and increasing anti-inflammatory factors (e.g., IL-10, TGF-β). This dual effect helps create a regenerative microenvironment that supports tissue healing [82,83]. Furthermore, exosomes enhance antioxidant defenses by delivering miRNAs and proteins that upregulate enzymes like superoxide dismutase and catalase, which neutralize ROS. Studies suggest that exosomal miR-126 and miR-210 promote angiogenesis and increase cellular resistance to oxidative stress, potentially improving blood flow and tissue repair in the urethral sphincter and pelvic floor muscles [84].

### 5.4. ECM Remodeling

**Figure 1:**
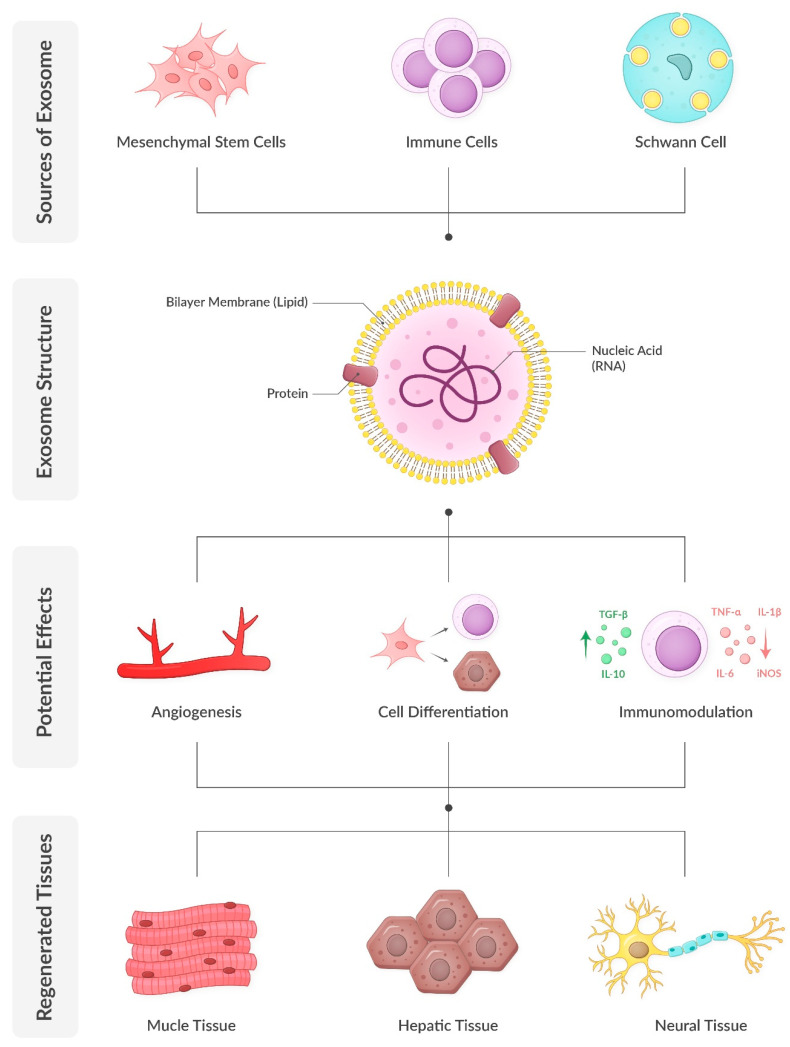
Exosome biology and regenerative potential.

SUI is characterized by a reduction in collagen and elastin within the ECM, leading to weakened urethral support. Exosomes derived from mesenchymal stem cells have been shown to enhance collagen synthesis by upregulating key genes, such as *COL1A1*, and inhibiting MMPs. For example, adipose-derived exosomes have demonstrated the ability to restore ECM integrity in fibroblasts from SUI patients by increasing collagen type I production while decreasing collagen degradation enzymes [85] (Figure 1).

## 6. Potential Integration of Exosome Therapy into Current Treatment Approaches

Exosome therapy has gained attention as an innovative regenerative approach that has the potential to enhance current treatment strategies by targeting underlying tissue degeneration.

### 6.1. Adjunct to PFMT or Rehabilitation

Exosomes have been shown to promote the regeneration of pelvic floor muscles by delivering bioactive molecules, such as growth factors and anti-inflammatory mediators, which could enhance the effectiveness of PFMT. The application of exosome-based therapies via intramuscular injections or topical administration may accelerate muscle repair, reduce fibrosis, and improve muscle contractility.

### 6.2. Combination with Pharmacotherapy

By modulating oxidative stress and inflammatory pathways, exosomes could enhance urethral sphincter function, potentially decreasing the need for pharmacological agents like duloxetine. Exosome therapy may also complement estrogen-based treatments by promoting ECM remodeling and vascularization, thereby improving urethral tissue integrity and function.

### 6.3. Adjunct to Surgical Interventions

The administration of exosomes in the perioperative period has demonstrated potential in improving tissue healing and reducing fibrosis, which could optimize long-term outcomes following surgical procedures such as mid-urethral sling placement. Additionally, exosome-based injections may serve as a superior alternative to traditional bulking agents by offering sustained tissue regeneration rather than temporary volume augmentation.

Over time, exosome therapy could prove to be a cost-effective solution if it delivers long-lasting regenerative benefits, minimizing reliance on continuous medication use and repeat surgical interventions. However, its widespread feasibility is contingent upon improvements in production efficiency, streamlined regulatory approval processes, and extensive clinical research to validate its effectiveness. As technological advancements drive down manufacturing costs and enhance accessibility, exosome therapy has the potential to emerge as a financially viable alternative to traditional treatments for SUI.

## 7. Preclinical Studies on Exosome Therapy for SUI

The initial application of regenerative therapy SUI can be traced back to the early 2010s [86,87]. Although early studies mainly focused on stem cells, growing interest in the paracrine effects of these cells led researchers to explore this area further.

Central to the pathology of SUI is ECM remodeling, characterized by reduced collagen and elastin concentrations, which weaken the supportive tissues. One such study is Liu et al.’s. The authors investigated the regulatory role of exosomes secreted by adipose-derived mesenchymal stem cells on collagen synthesis in fibroblasts of women with SUI in vitro [85]. Their findings demonstrated that exosomes increased the synthesis of collagen type 1 by upregulating the expression of the col1a1 gene, which is responsible for collagen type 1 production. Additionally, these exosomes modulated the expression of MMPs, showing a decreased expression of MMP-1 and MMP-3, while their inhibitory genes (TIMPs) were upregulated. The MMP gene family includes various collagenase enzymes that degrade collagen; thus, downregulation of these genes by exosomes results in reduced collagen degradation. The researchers concluded that since SUI is associated with up to a 60% reduction in collagen concentration [88], exosomes may offer therapeutic benefits by enhancing collagen production and reversing ECM deterioration associated with SUI.

Building on this foundation, to investigate the pathway through which exosomes enhance ECM remodeling and collagen synthesis, Wang et al. conducted an in vitro study on the effects of EVs from adipose-derived mesenchymal stem cells (ADSCs) on primary fibroblasts and satellite cells in a SUI model [89]. In fibroblasts, RT-qPCR and Western blot assays revealed that the expression levels of elastin, collagen I, and collagen III increased after treatment with ADSC-EVs. Additionally, in satellite cells, which are key to muscle tissue repair, immunofluorescence and Western blot assays showed that the expression of Pax7 and MyoD was elevated following treatment with ADSC-EVs, whereas these levels were significantly reduced in the control group. This suggests that ADSC-EVs play a role in collagen synthesis and satellite cell activation. ADSC-EVs are known to carry miR-93 [90], a miRNA previously implicated in ECM remodeling in SUI [91]. The authors investigated the expression of miR-93 before and after treatment. As expected, miR-93 levels were significantly reduced in SUI fibroblasts and satellite cells, but notably increased after treatment with ADSC-EVs. Furthermore, the expression of coagulation factor III (F3) was reduced after treatment, suggesting that miR-93 from ADSC-EVs may exert its regenerative effects by downregulating this gene.

In parallel, Zhang et al. investigated the effects of bone marrow mesenchymal stem cell-derived small extracellular vesicles (BMMSC-sEVs) on ECM remodeling in SUI, both in vivo and in vitro [92]. Their study also suggested that BMMSC-sEVs improved ECM components by promoting the synthesis of elastin, collagen I, and collagen III, as well as enhancing muscle tissue by increasing muscle fiber integrity and reducing atrophy. Detailed analysis revealed that these sEVs exerted their effects by transferring miR-328a-3p, which downregulated the Sirt7 pathway. This, in turn, activated and upregulated the TGF-β1/Smad signaling pathway, known for its crucial role in ECM remodeling and maintenance [93]. Overall, the administration of BMMSC-sEVs led to improved function of the damaged urethral sphincter, as evidenced by significantly increased leak point pressure (LPP) values, highlighting the potential of BMMSC-sEVs in the treatment of SUI.

**Figure 2:**
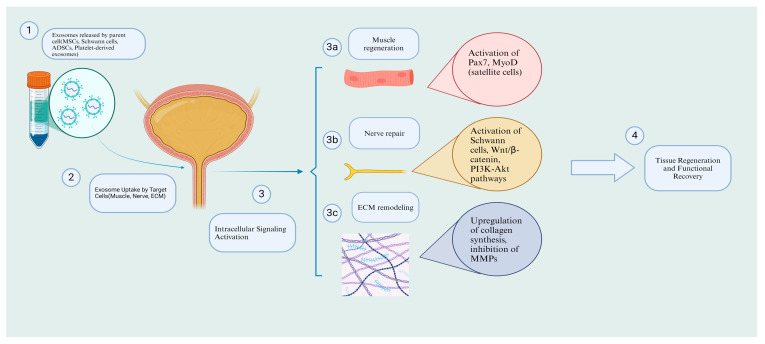
Mechanisms of exosome therapy in SUI: cellular and molecular pathways. Created in https://BioRender.com.( accessed on 16 February 2025).

Beyond ECM remodeling, exosomes have shown promise in muscle regeneration, a key factor in SUI recovery, as demonstrated in a study by Wu et al. Wu and colleagues observed that rats with SUI, when treated with urine-derived stem cell exosomes, exhibited significant improvements compared to the control group [94]. The study showed that exosomes activated and promoted satellite cells by increasing the expression of Pax3, Pax7, MyoD+, and cyclin B/D1/E, which in turn enhanced myotube formation, reduced secondary necrosis of muscle fibers, and ultimately led to normal muscle regeneration. Consequently, rats treated with exosomes displayed significant urodynamic improvements compared to the control group. Interestingly, when these parameters were compared to healthy rats without SUI, no significant differences were observed, suggesting that exosomes could potentially achieve full recovery in SUI-related defects. Similarly, Li et al. explored the therapeutic potential of exosomes derived from silent mating type information regulation 2 homolog 1 (SIRT1)-overexpressing human BMSCs on the pubococcygeus muscle in a rat model of SUI [95]. Their findings also revealed the activation of satellite cells through increased expression of Pax3, Pax7, and cyclin B/D1/E. Moreover, the differentiation and proliferation of these cells were enhanced by the upregulation of MHC, MyoD, and Myosin. The ERK pathway, which plays a crucial role in satellite cell differentiation, was also found to be elevated, as confirmed by Western blot analysis. These cellular and molecular events ultimately led to improved muscle fiber structure and reduced atrophy, which were validated by enhanced urodynamic outcomes (Figure 2).

In another study, Rolland et al. examined the effect of human platelet-derived exosomes (PEPs) on skeletal muscle regeneration in SUI, both in vitro and in vivo [71]. They observed that human skeletal muscle myoblasts (HSMMs) co-cultured with PEPs exhibited dose-dependent growth. Specifically, PEP concentrations greater than 1.25 × 10^10^ exosomes/mL resulted in 90% growth of myoblasts compared to 50% growth when cultured with standard growth media containing 10% FBS. Previous research on skeletal muscle regeneration has indicated that the upregulation of MyoD1, Myf5, and Pax7 during the early stages of regeneration is associated with the activation of the NF-κB pathway [96,97]. Interestingly, in this study, it was reported that when resveratrol, a known inhibitor of the NF-κB pathway, was administered alongside PEPs, there was a dose-dependent decrease in muscle regeneration compared to those treated with PEPs alone. This finding underscores the importance of the NF-κB pathway in muscle regeneration when PEPs are used. Additionally, PDL-1 expression was observed to be upregulated, leading to a macrophage environment dominated by M2-type macrophages, which are essential for maintaining proper homeostasis during muscle regeneration [98,99]. The in vivo experiments included two sets of animals. First, the researchers evaluated the effect of these exosomes in rats with defects in the latissimus dorsi muscle, which led to significant muscle regrowth. Finally, they administered the exosomes to a porcine SUI model and assessed sphincter function using urethral pressure profiling. Notably, the treatment group showed significant improvement, with no leakage observed at the end of the study, further emphasizing the potential of exosomes for full recovery in SUI.

Although the regenerative effects of M2 macrophages have been well-documented, M1 macrophages have also shown regenerative potential through a different pathway [100,101]. A notable study by Cheng et al. investigated the effects of M1-type macrophage exosomes on a mouse model of SUI induced by labor trauma [102]. Their research demonstrated that administering these exosomes led to significant improvements in urodynamic parameters, including abdominal leak point pressure (ALPP) and maximum bladder volume (MBV). Moreover, histological analysis revealed that these exosomes could prevent secondary necrosis of damaged muscle fibers, reduce nuclear migration within muscle fibers, maintain the structural integrity of muscle bundles, and promote normal muscle regeneration, effectively reversing the effects of labor-induced SUI.

Another dimension of exosome therapy involves the regeneration of peripheral nerves, specifically the pudendal nerve, which play a critical role in SUI pathology. Huang et al. investigated the regenerative effects of SC-derived exosomes on a SUI model in rats with damaged dorsal root ganglion (DRG) cells, which are known to play a role in peripheral nerve diseases [103]. The study demonstrated that SC-derived exosomes had protective effects on DRG cells following mechanical damage. These exosomes were able to stimulate cell proliferation, promote the transition of the cell cycle to the G2 phase, and inhibit cell apoptosis, thereby facilitating nerve regeneration. Previous research has established that the Wnt/β-catenin signaling pathway is closely associated with nervous system development, stem cell proliferation and differentiation, and axon guidance [104,105,106]. Therefore, the authors hypothesized that the regenerative effects of these exosomes might be mediated through this pathway. To test this hypothesis, they administered the exosomes in combination with XAV939, an inhibitor of the Wnt/β-catenin signaling pathway. The results showed that the regenerative effects of the exosomes were significantly inhibited, underscoring the importance of the Wnt/β-catenin pathway in this process. However, the researchers noted that this is likely not the only pathway involved, and further research is needed to identify additional mechanisms. Notably, miRNA-133b in exosomes derived from pluripotent mesenchymal stromal cells and miRNA-219 in serum-derived exosomes have also been shown to be effective against damaged nerve cells [107,108], highlighting promising avenues for future research. Zhou et al. also examined the effects of RSC96 SC-derived exosomes on damaged DRG cells, with findings consistent with those of Huang et al.’s study [103,109]. As previous research has highlighted, axonal regeneration is crucial for nerve repair, largely driven by SC activity and signaling [110]. Consequently, the authors used these exosomes to treat SUI in rats with damaged DRG cells. Their results showed that the proliferation rate of the damaged DRG cells increased significantly, returning to normal levels after incubation with the exosomes. Additionally, flow cytometry revealed decreased apoptosis and reduced levels of SA-β-gal, a widely recognized biomarker for cellular senescence, leading to significant neural repair. In conclusion, the authors suggested that exosomes can promote nerve regeneration by inducing proliferation, inhibiting apoptosis, and delaying senescence.

Ni et al. investigated the effects of human ADSC exosomes on SUI both in vivo and in vitro, using a model that involved dual injury to the pudendal nerve and the muscular compartment [2]. They also compared the therapeutic efficacy of exosomes with that of their progenitor stem cells. Although the difference in treatment outcomes was not significant, exosome therapy yielded better therapeutic results. In vitro, a CCK-8 assay on skeletal and glial cells demonstrated significant dose-dependent cellular proliferation in both skeletal and SC lines, with the strongest proliferation observed at a concentration of 5 µg/mL. This dosage was subsequently used in female rats with defects in both the skeletal and nerve compartments. The in vivo study showed enhanced muscle and nerve regeneration, as exosome-treated groups exhibited increased muscle and nerve fiber growth, along with reduced atrophy. This finding was consistent with functional assessments, as cystometrography and LPP values showed significant improvement. Notably, the therapeutic effects of the exosomes persisted and even increased over time. To elucidate the underlying mechanisms, proteomic analysis using GO and KEGG pathways revealed that the PI3K-Akt, Jak-STAT, and Wnt pathways—known to be associated with muscle and nerve proliferation and regeneration—were upregulated, highlighting their role in these processes.

Detailed information regarding preclinical studies is available in Table 2.

The extensive involvement of exosomes in tissue repair and regeneration highlights their adaptability and therapeutic potential. By supporting crucial processes such as angiogenesis, cellular differentiation, and immunomodulation, exosomes offer an exciting approach to healing. Their capability to regulate the inflammatory response and foster an environment supportive of tissue repair further emphasizes their promise as an innovative therapeutic option. As ongoing research continues to reveal the mechanisms and advantages of exosome therapy, its application in treating conditions like SUI appears increasingly promising. Therefore, exosomes stand at the forefront of regenerative medicine, presenting new possibilities for effective and targeted treatment.

## 8. Challenges and Future Directions

Several critical challenges must be addressed to facilitate the clinical translation of exosome therapy for SUI. A key concern is the precise mechanism by which exosomes contribute to muscle and nerve regeneration, particularly their interactions with signaling pathways such as Wnt/β-catenin and PI3K-Akt. Additionally, while preclinical studies have demonstrated promising short-term outcomes, rigorous long-term assessments are necessary to establish the therapy’s safety and efficacy. Optimizing treatment parameters, including dosage, frequency of administration, and the choice between single or multiple injections, remains an area requiring further investigation. Current evidence suggests that multiple administrations may enhance therapeutic benefits; however, the optimal regimen must be determined based on factors such as the exosome source, concentration, and delivery method. Clinical trials are essential to develop standardized, evidence-based treatment guidelines.

Furthermore, exploring the combination of exosome therapy with other regenerative approaches or current treatment methods, such as combining conservative treatments with exosome therapy, could enhance the long-term efficacy of current methods, ultimately resulting in more comprehensive and effective treatments.

Currently, there is no standardized protocol for exosome isolation due to the diversity of available techniques. Exosome characterization remains inherently complex, as each isolation and characterization method has distinct advantages and limitations. Current research focuses on identifying a universal set of exosomal marker proteins common to all cell types. Furthermore, studies have shown that different isolation methods applied to the same cell type can yield distinct proteomic profiles, adding another layer of complexity [112]. Exosome composition is highly sensitive to the conditions of the culture medium in which they are secreted, leading to variability even among exosomes derived from the same cell source. This contributes to batch-to-batch inconsistencies, posing a significant challenge for reproducibility. To address these issues, the European Network on Microvesicles and Exosomes in Health and Disease, along with the International Society for Extracellular Vesicles, has published standard operating procedures (SOPs) outlining regulatory guidelines for exosome isolation, characterization, functionality assessment, and manufacturing to facilitate their therapeutic applications. Despite the growing interest in exosome-based therapies and promising market projections, clinical translation remains hindered by scientific and regulatory challenges. These include the absence of universally accepted isolation protocols, difficulties in establishing pharmacokinetics and therapeutic efficacy, and the need to navigate FDA guidelines [113,114].

Another challenge that needs to be addressed is exosome functionality evaluation before administration. While for stem cell therapy methods such as cell viability assays are available to determine their function, in exosomes, a standardized protocol is still lacking. Exosomes are mainly characterized by their morphology, physical attributes, surface markers, and cargo content. Methods such as electron microscopy, flow cytometry, nanoparticle tracking analysis, and dynamic light scattering have previously been employed to achieve this [13,112]. However, a unified method to achieve optimal functionality testing before administration is still lacking and needs to be addressed in future studies.

Exosomal storage is also another point that needs to be critically investigated in the future, as preserving exosomes is challenging. They remain most stable when stored long-term at approximately −80 °C; however, this low temperature can impair translational activity. Freeze-drying, which involves vacuum-assisted removal of water from a frozen sample, offers a potential solution. Despite its benefits, freeze-drying may alter the number, stability, and biological activity of exosomes. Additionally, exosomes have a tendency to aggregate during the freeze-drying process, potentially reducing their functionality. To counteract this, stabilizing agents such as starch or glucose can be incorporated during cryopreservation [115,116].

Exosomes, despite being cell-free therapeutic agents, present specific safety concerns. Notably, a primary component of exosomes is miRNAs, some of which exhibit oncogenic properties that may induce excessive proliferation and differentiation in target cells, potentially leading to tumorigenesis. The current understanding of this issue is limited, necessitating future studies to rigorously examine the influence of originating cells and isolation methods on the presence of these components in exosomes. Additionally, due to their small size, comparable to certain pathogens such as viral particles (e.g., retroviruses), exosomes are susceptible to contamination. Therefore, stringent production and manufacturing procedures under strict sterile protocols are essential to prevent contamination [117]. Recent advancements in bioengineering have enabled the modification of exosomes to enhance target specificity and therapeutic efficacy by engineering their surface markers and cargo composition. This approach holds promise for reducing off-target effects and improving treatment outcomes. However, the inherent heterogeneity of exosomes remains a significant obstacle, underscoring the need for standardized profiling and quality assessment protocols [118].

Future research should also prioritize optimizing exosome delivery methods to target tissues, thereby enhancing therapeutic efficacy. Determining the most effective delivery method for exosome therapy in SUI is crucial. Periurethral injections offer targeted delivery to the affected area, potentially enhancing therapeutic efficacy. However, the optimal delivery method may vary depending on individual patient factors and requires further investigation. While animal models provide valuable insights, their translatability to human applications remains uncertain. Species-specific differences in immune responses, ECM composition, and regenerative capacity may impact therapeutic efficacy. For example, variations in urethral sphincter structure and muscle regeneration rates between rodents and humans could influence treatment outcomes. Additionally, the pharmacokinetics, biodistribution, and long-term integration of exosome therapy in human tissues require further investigation. Given these discrepancies, clinical trials are essential to validate the safety, efficacy, and optimal dosing strategies of exosome therapy in human patients. To date, no human studies have been conducted on this matter due to challenges such as regulatory hurdles, scalability issues, and cost considerations, underscoring the need for clinical research to bridge the gap between preclinical evidence and practical application. However, in the field of Urology, a number of clinical trials have been carried out, primarily focusing on the diagnostic potential of exosomes in prostate cancer with promising outcomes (NCT03031418, NCT04053855 at clinicaltrial.gov, accessed on 16 February 2025). Moreover, a recent study is currently recruiting patients to treat erectile dysfunction using exosomes (NCT06605508 at clinicaltrial.gov, accessed on 16 February 2025), highlighting their advancement and potential in this field. Addressing these challenges will be crucial for advancing exosome therapy from experimental research to clinical practice.

## 9. Conclusions

SUI is a challenging and often debilitating condition marked by the involuntary leakage of urine during everyday activities such as coughing, sneezing, and physical exertion. It is particularly prevalent among post-menopausal women with a history of vaginal delivery. Despite the availability of treatment options, high recurrence rates and the risk of complications associated with surgical interventions emphasize the need for novel and more effective therapeutic approaches.

Regenerative medicine, specifically the use of exosomes, offers a promising avenue for addressing the underlying causes of SUI. Exosomes have shown potential in promoting tissue regeneration, reducing fibrosis, and enhancing repair mechanisms, suggesting that they could serve as a viable alternative or adjunct to current SUI treatments. Importantly, exosome-based therapies could reduce reliance on invasive surgical interventions, ultimately improving patients’ quality of life. Although current research is primarily in the preclinical stage, the encouraging results to date suggest a potential path toward more effective and lasting treatments for SUI. However, to translate this promising therapy into clinical practice, further research is critical. Well-designed preclinical studies should continue to elucidate the precise mechanisms underlying exosome-mediated repair, while rigorous clinical trials are necessary to evaluate their safety, efficacy, and long-term outcomes. Standardization of exosome isolation, characterization, and delivery methods will also be essential to ensure reproducibility and optimize therapeutic potential, with the ultimate aim of restoring normal continence.

## Figures and Tables

**Table 1:** Current treatment modalities for SUI: mechanisms, benefits, and limitations.

Treatment	Description	Limitations and Success Rate (SR)
Pelvic Floor Muscle Training (PFMT) [24]	PFMT involves a regimented set of exercises aimed at strengthening the pelvic floor muscles, enhancing muscle strength, endurance, and control. It is frequently recommended as a first-line therapy, particularly for postpartum women.	Achieving efficacy requires consistent adherence and accurate technique, posing a challenge for some patients. Additionally, the therapeutic outcomes can vary widely, with some women experiencing minimal improvement or a recurrence of symptoms 50–70% SR
Pessaries [25]	Pessaries are intravaginal devices that support the vaginal walls, thereby reducing SUI symptoms. They are a conservative treatment option for those seeking to avoid or postpone surgery.	Pessaries necessitate regular maintenance and monitoring by healthcare providers to prevent complications such as vaginal erosion or infection. They may not provide sufficient relief for women with severe incontinence 76% SR
Mid-Urethral Sling (MUS) [26,27]	The MUS procedure is a minimally invasive surgery that places a synthetic mesh sling under the urethra for added support and reduced urine leakage. Variants include tension-free vaginal tape and trans-obturator tape.	Potential complications include mesh erosion, pain, and infection, which may require additional surgeries. The procedure is contraindicated in patients with a history of pelvic surgery or radiation therapy 80–94% SR
Autologous Pubovaginal Slings (PVS) [28,29]	This surgical intervention uses the patient’s tissue, typically from the rectus fascia or thigh, to create a sling for urethral support, making it suitable for patients with recurrent or severe SUI.	Compared to synthetic slings, autologous PVS is associated with longer recovery times and higher surgical morbidity. Postoperative complications may include voiding dysfunction and the need for catheterization 84–96% SR
Urethral Bulking Agents [30]	Urethral bulking involves the injection of materials around the urethra to enhance closure and prevent leakage. Common agents include silicone particles and carbon spheres.	This approach is generally less effective than surgical alternatives and is associated with a higher recurrence rate of symptoms. Repeat treatments are often necessary as the bulking agents’ effects wane over time 46–57% SR
Artificial Urethral Sphincter (AUS) [31,32]	The AUS is a mechanical device implanted to manage urine flow, primarily used in women with severe intrinsic sphincter deficiency unresponsive to other treatments.	AUS implantation carries a high risk of complications, including device malfunction, erosion, and infection. The complex nature of the surgery entails a significant recovery period and the potential for additional operations 79–86% SR
Bladder Neck Incision (BNI) [33]	BNI is a surgical procedure that creates an incision at the bladder neck to enhance urinary flow, typically used for SUI resulting from bladder outlet obstruction.	The procedure carries a risk of recurrent incontinence and complications such as vesicovaginal fistula formation. The limited data on long-term efficacy make it a less commonly selected option 72–84.5% SR
Colposuspension (Burch Procedure) [34]	This surgical technique involves the suspension of the bladder neck using sutures to elevate and support it.	Colposuspension is more invasive than sling procedures and is associated with risks such as infection, bleeding, and a longer recovery period 82.9–94.83% SR
Pharmacotherapy [35]	Pharmacological agents, such as duloxetine, are used to enhance urethral sphincter muscle tone. Research indicates that the α2-AR antagonist imidazoxan can augment the effects of duloxetine on the sneeze-induced urethral continence reflex in rats.	Side effects, including nausea, dry mouth, and fatigue, can limit the acceptability of these medications. The variability in patient response further complicates their use

**Table 2:** Preclinical studies on the therapeutic potential of exosomes in SUI.

Author	Exosome or Ev Source	Exosome Isolation Method	Type of Study	Animal/Model	Method of Injection	Dosage	Detection Method	**Results**
Zhou et al. 2018 [109]	RSC96 Schwann cell exosomes	ExoQuick-TC™ exosome precipitation solution (EXOTC10A-1) (System Biosciences, Palo Alto, CA, USA).	In vitro	Cyclic mechanical strain (CMS) of DRG cells	Co-cultured	20 µg/mL concentration	CCK-8 assay/SA-b-gal staining/Flow cytometry^1^	Promotion of cell proliferation and significantly inhibited apoptosis and senescence
Liu et al. 2018 [85]	Adipose-Derived Mesenchymal Stem cell exosomes	Differential centrifugation	In vitro	Vaginal fibroblasts from women with SUI	Co-cultured	Dosage was not specified, cultured for 48 h	qRT-PCR/Western blot	Increased collagen synthesis via upregulation of col1a1 gene/decreased collagen degradation via upregulation of TIMP-1 and TIMP-3 and downregulation of MMP-1 and MMP-2
Ni et al. 2018 [2]	Adipose-Derived Mesenchymal Stem cell exosomes	Ultrafiltration	In vitro/In vivo	L6 rat skeletal muscle cells and RSC96 rat Schwann cells/rats with PNT and VD ^2^	Co-cultured/Peripheral urethra injection	control medium containing exosomes at three different concentrations (0.05, 0.5, and 5 μg/mL) for 72 h /50 μg exosomes suspended in 50 μL saline for injection	CCK-8 assay/CMG and LPP/H&E ^3^ and Masson trichrome/immunofluorescent staining/LPP	Enhanced growth of skeletal muscle and Schwann cell lines in a dose-dependent manner in association with PI3K-Akt, Jak-STAT, and Wnt pathways/higher bladder capacity and LPP, more striated muscle fibers and peripheral nerve fibers in the urethra
Wu et al. 2019 [94]	Urine-Derived Stem cell exosomes	Differential centrifugation	In vivo	Rats/vaginal balloon inflation	local injection in and around the Pubococcygeus muscle	1 mL of 1 × 10^10^ particles/mL Exosomes	CCK-8 assay/immunofluorescence staining/Western blot/RT-qPCR/H&E and Masson trichrome/EdU ^4^ cell proliferation assay	Significantly improved urodynamic parameters/activation, and proliferation of satellite cells through upregulation of Ras-ERK signaling
Zhang et al. 2020 [92]	BMMSC-Derived sEV	Differential centrifugation	In vitro/In vivo	Primary fibroblasts/rats with transabdominal urethrolysis	Co-cultured/trans-urethral wall injection	different concentrations of BMMSC-sEV (1 μg/mL, 5 μg/mL, 10 μg/mL, or 20 μg/ ml) for co-culture/ 100 μg BMMSC-sEV once a week for 5 weeks	Western blot/LPP/H&E, EVG ^5^, and Sirius red staining/Immunofluorescence staining/miRNA sequencing/Dual-luciferase reporter assay/Cell transfection	Transferred miR-328a-3p of BMMSC-sEV promoted ECM remodeling of damaged urethral sphincters by inhibiting SirtT7 and activating TGF-β1 signaling pathway/significantly improved LPP values
Wang et al. 2021 [89]	Adipose-Derived Mesenchymal Stem Cells EV’s	Differential centrifugation	In vitro	Primary satellite cells/primary fibroblasts	Co-cultured	ADSC conditioned medium with 30 mg ADSCs-EVs dissolved in 100 mL PBS	Immunofluorescence Staining/RT-qPCR/Western blot/Dual-Luciferase Reporter Gene Assay	EVs increased Elastin, Collagen I, and Collagen III in fibroblasts, resulting in ECM remodeling and increased Pax7 and MyoD in satellite cells through transferring miR-93 and suppressing F3 pathway
Li et al. 2021 [95]	SIRT1-Overexpressing Bone Marrow Mesenchymal Stem Cells exosomes	Differential centrifugation	In vivo	Rats/VD	local injection in and around the Pubococcygeus muscle	1 mL exos (1 × 10^10^ particles/mL)	CCK-8 assay/Western blot/Immunofluorescence staining/RT-qPCR/ ALPP/MBV	SIRT1/exos exert their therapeutic effect through the ERK pathway, leading to activation and proliferation of satellite cells, resulting in repaired muscular fibers/significantly improved urodynamic parameters such as ALPP and MBV
Cheng et al. 2021 [102]	M1-type Macrophage exosomes	Differential ultracentrifugation	In vitro/In vivo	CMS of C2C12 myoblasts/rats with vaginal balloon expansion	Co-cultured/local injection in and around the Levator ani muscle	1 × 10^10^ particles/mL, 1 mL of exosomal solution was cultured for 24 h/ 1 mL exos (1 × 10^10^ particles/mL)	ALPP/MBV/H&E/CCK-8 assay/SA-b-gal staining/Flow cytometry ^1^ (PE/7-AAD staining)/EdU cell Proliferation/	M1-Exo helps in the functional and anatomical recovery of SUI mice/Promoting proliferation, inhibiting apoptosis and delaying senescence in myoblasts
Huang et al. 2021 [103]	RSC96 Schwann cell exosomes	ExoQuick-TC Exosomes Isolation Kit (EXOTC10A-one, system biosciences, SBI, USA)	In vitro	CMS of DRG cells	Co-cultured	20 μg of exosomes for 24 h	CCK-8 assay/cell cycle and apoptosis detection kit/Annexin V and PI staining/Flow cytometry/Western blot	Schwann cell exosomes stimulate cell proliferation, promote the transition of the cell cycle to the G2 phase, and inhibit cell apoptosis, thereby facilitating nerve regeneration through Wnt/β-catenin signaling pathway
Jiang et al. 2021 [111]	Bone Marrow Stem cells secretomes	Differential centrifugation	In vitro/In vivo	Primary fibroblasts/rats with VD	Co-cultured/Periurethral injection	0.4 mL of Cell culture-conditioned medium	CCK-8 assays/cell Migration and Wound-scratch assay/Western blot/RNA sequencing/Immunofluorescence staining/LPP/IHC ^6^/Histology/ JAK2/STAT4 verification	BMSC-CM facilitates the proliferation, migration, and collagen production in AVW ^7^ fibroblasts via the JAK2/STAT4 pathway to improve urinary incontinence and accelerate the regeneration of AVW collagen fibers in a SUI rat model
Rolland et al. 2022 [71]	Human Platelet-derived exosomes	Differential centrifugation	In vitro/In vivo	Skeletal muscle satellite cells/pigs with full thickness lesion (extending the urethral wall and overlying the urethral sphincter)/rats with latissimus dorsi volumetric muscle loss	Co-cultured/Periurethral injection	Increasing concentrations of PEP ranging from 1.25 × 10^11^ exosomes/mL to 5 × 10^11^ exosomes/mL/ 100 μL of DiR labeled PEP (5 × 10^12^ exosomes/mL).	Western blot/ AFM ^8^ and SP-IRIS ^9^ analysis/scratch wound assay/IncuCyte Live-Cell and Chemotaxis module analysis/Exosome release assay/Medspira catheter to asses’ urethral pressure/portable mCompass Anorectal Manometry system/Histology/	Significant growth of human skeletal muscle myoblasts through NF-κB and PDL-1 pathway in a dose-dependent manner/muscle regeneration in rat model/significantly improved urethral pressure values with no leakage

1: Hoechst 33258 (blue) fluorescence nucleic acid staining using flow cytometry 2: VD: vaginal dilation, PNT: pudendal nerve transection 3: hematoxylin and eosin 4: 5-ethynyl-2′-deoxyuridine 5: Elastic van Gieson to detect elastin expression 6: immunohistochemistry 7: anterior vaginal wall 8: Atomic-force microscope 9: single-particle interferometric reflectance imaging sensing.
